# Prediction of outcomes in patients with severe aortic stenosis after multiple valve surgery

**DOI:** 10.1186/1749-8090-10-S1-A241

**Published:** 2015-12-16

**Authors:** Vladislav Podpalov, Vasily Sevrukevitch, Svetlana Kurganovich, Siarhei Spirydonau, Irina Russkih, Marina Kolyadko, Alexander Deev, Yuri Ostrovsky

**Affiliations:** 1 Republican Scientific and Practical Center of Cardiology, Minsk, 220036, The Republic of Belarus; 2 National Research Center for Preventive Medicine, Moscow, 101990, Russian Federation

## Background/Introduction

Aortic stenosis (AS) occupies the first place in the list of diseases leading to valve replacement surgery. Biomarker NT-pro BNP was proved to have predictive power in people with coronary artery disease. However the relationship between NT-proBNP level and outcomes after single valve replacement and multiple valve surgery for severe AS is still controversial.

## Aims/Objectives

To analyze the role of NT-proBNP in prediction of outcome after multiple valve surgery for severe AS.

## Method

Among all patients treated at the Center during March 2012 - September 2013 there were 49 patients with severe AS and secondary mitral and tricuspidal regurgitation. They were included into the prospective study. Surgical treatment consisted of aortic valve replacement (AVR) in combination with atrioventricular valves repair. Follow-up period was 1 year. Patients' data, including NT-proBNP values, were collected preoperatively and 1 year after surgery.

## Results

Average patients' age was 63,2 ± 10,3 years, proportion of men - 53,1%. Postoperative 30-day mortality was 2,0%, 1-year mortality - 8,2%. The average NT-BNP level was 8060,20 ± 7286 pg/ml preoperatively and 870,1 ± 1591 after operation (p < 0,001). Using multivariate regression model a significant independent negative relationship was obtained between NT-proBNP level and the following parameters: aortic valve effective orifice area (p < 0,001), left ventricular ejection fraction (p = 0,001). Preoperative level of NT-proBNP didn't correlate with 1 year survival (p = 0,36). However a significant negative association was found between preoperative NT-proBNP values and risk factor profile of 5-year survival (p = 0,003) described in previous studies and consisted of age, sex, aortic valve peak systolic gradient, LVEF, pulmonary artery systolic pressure and left ventricular myocardial mass. When NT-proBNP values increased probability of 5-year survival.

## Discussion/Conclusion

Biomarker NT-proBNP can predict 5-year survival in patients with severe AS after multiple valve surgery. In Further Studies with greater amount of patients this relationship should be detailed.

